# Beyond the Block: Cariprazine in Treatment-Resistant Depression

**DOI:** 10.1192/j.eurpsy.2026.11425

**Published:** 2026-07-31

**Authors:** T. Grahovac Juretić, A. Mančić, K. Ružić, M. Letica-Crepulja, E. Dadić-Hero

**Affiliations:** Department of psychiatry, University Hospital Centre Rijeka, Rijeka, Croatia

## Abstract

**Introduction:**

Treatment-resistant depression (TRD) remains one of the most challenging conditions in psychiatry, particularly when accompanied by psychotic features. Patients often undergo multiple treatment strategies, including various classes of antidepressants, augmentation with antipsychotics, and non-pharmacological interventions, yet remission is frequently incomplete. Cariprazine, a dopamine D3/D2 receptor partial agonist, has emerged as a potential therapeutic option for resistant cases, especially when negative or residual symptoms predominate.

**Objectives:**

To present the clinical course of a patient with long-standing treatment-resistant depression and to evaluate the therapeutic impact of cariprazine following multiple unsuccessful treatment strategies.

**Methods:**

We report on a 65-year-old married woman, mother of three adult children, with a 20-year history of severe depressive episodes with psychotic features. She had multiple hospitalizations in psychiatric institutions and was treated with numerous antidepressant combinations (e.g., venlafaxine + mirtazapine, bupropion + mirtazapine, fluvoxamine + reboxetine, mirtazapine + paroxetine) alongside both typical and second-generation antipsychotics. In addition, she participated in continuous group psychotherapy. Symptom improvement was partial, with residual negative symptoms persisting. The patient also underwent two courses of transcranial magnetic stimulation (TMS) using the standard protocol for depression, each producing temporary improvement lasting up to six months.

**Results:**

Introduction of cariprazine, titrated to 4.5 mg daily, resulted in a gradual reduction of persistent negative symptoms. Over time, the patient achieved not only symptomatic remission but also partial functional remission. She currently functions satisfactorily across social, occupational, and personal domains, representing a significant improvement compared to prior treatment phases.

**Conclusions:**

This case highlights the potential role of cariprazine in addressing residual symptoms of treatment-resistant depression, particularly where previous pharmacological and non-pharmacological strategies yielded only partial or temporary effects. Further studies are warranted to explore whether cariprazine can consistently provide both symptomatic and functional benefits in TRD patients.

**Disclosure of Interest:**

None Declared

